# Saving Claire: Effectiveness of an Innovative Approach to Preventing Falls in the Community

**DOI:** 10.1093/geroni/igaf122.1755

**Published:** 2025-12-31

**Authors:** Sara Pappa, Cathy Elrod, Mahederemariam Dagne, Elizabeth Terhune, Erin Staker, Patricia Heyn

**Affiliations:** Marymount University, Arlington, Virginia, United States; Marymount University, Arlington, Virginia, United States; Marymount University, Arlington, Virginia, United States; Marymount University, Arlington, Virginia, United States; Center for Optimal Aging, Arlington, Virginia, United States; Marymount University, Arlington, Virginia, United States

## Abstract

Falls are a major concern for older adults. According to the Centers for Disease Control and Prevention (CDC), about 3 million emergency room visits among older adults are due to fall injuries and over 300,000 hospitalizations are due to hip fractures. Several community-based interventions are being implemented yearly including evidence-based falls prevention programs. Additionally, several behavioral interventions are also implemented to increase awareness of falls and fall injuries. One novel approach to community-based falls prevention efforts is the Saving Claire (SC) project, an event that combines storytelling, a documentary film viewing, and an expert-led panel discussion. We implemented the SC project in the Northern Virginia region starting in September 2023. The expert panel comprised members of the community, including a physical therapist, an occupational therapist, a home modification expert, and other health professionals to provide advice and answer questions on falls prevention strategies. We also provided an optional survey to capture program effectiveness starting in April 2024 to evaluate the effectiveness and use of the project as a falls prevention education tool. We collected 306 surveys between April - October. Our preliminary data analysis showed that the majority of participants reported that the film and panel increased their understanding and awareness of falls prevention strategies. To date, the SC project has reached over 1,000 individuals. This study takes a deeper dive into the data, including participants’ intent to have a home safety check; get their hearing, vision, medications, and balance evaluated; and participate in an exercise program.

